# Onset of hypothyroidism after total laryngectomy: Effects of thyroid gland surgery and preoperative and postoperative radiotherapy

**DOI:** 10.1002/hed.26048

**Published:** 2019-12-13

**Authors:** Robert E. Plaat, Boukje A. C. van Dijk, Anneke C. Muller Kobold, Roel J. H. M. Steenbakkers, Thera P. Links, Bernard F. A. M. van der Laan, Boudewijn E. C. Plaat

**Affiliations:** ^1^ Department of Otorhinolaryngology, Head and Neck Surgery University of Groningen, University Medical Center Groningen Groningen The Netherlands; ^2^ Department of Otorhinolaryngology, Head and Neck Surgery Medical Center Leeuwarden Leeuwarden The Netherlands; ^3^ Department of Epidemiology University of Groningen, University Medical Center Groningen Groningen The Netherlands; ^4^ Department of Research and Development Netherlands Comprehensive Cancer Organization (IKNL) Utrecht The Netherlands; ^5^ Department of Laboratory Medicine University of Groningen, University Medical Center Groningen Groningen The Netherlands; ^6^ Department of Radiotherapy University of Groningen, University Medical Center Groningen Groningen The Netherlands; ^7^ Department of Endocrinology University of Groningen, University Medical Center Groningen Groningen The Netherlands

**Keywords:** hemithyroidectomy, hypothyroidism, radiotherapy, total laryngectomy

## Abstract

**Background:**

To determine time of onset and risk of hypothyroidism after total laryngectomy (TL) with and without (hemi)thyroidectomy in relation to treatment regimen, that is, preoperative radiotherapy (RT‐TL), postoperative radiotherapy (TL‐RT), and postoperative re‐irradiation (RT‐TL‐RT).

**Methods:**

Retrospective review of 128 patients treated by RT‐TL (51 patients), TL‐RT (55 patients), and RT‐TL‐RT (22 patients). Risk of hypothyroidism was determined by multivariable Cox regression analysis and euthyroid survival was calculated using Kaplan‐Meier method.

**Results:**

Hypothyroidism developed in 69 (54%) patients. The median onset of hypothyroidism was later (*P* < .01) and the risk of hypothyroidism was lower (hazard ratio 0.49; *P* = .014) in the TL‐RT group compared to both other treatment regimens. Euthyroid survival did not differ between the treatment regimens. Two years euthyroid survival was 24% with and 61% without (hemi)thyroidectomy (*P* < .001).

**Conclusions:**

Patients treated with TL‐RT have later onset of hypothyroidism. Higher risk for hypothyroidism is associated with salvage TL after radiotherapy and (hemi)thyroidectomy.

## 1 INTRODUCTION

Treatment options for laryngeal and hypopharyngeal carcinoma depend on tumor stage and focus on total cure with the preservation of a functional larynx. Transoral laser surgery for early laryngeal cancer or (chemo)radiotherapy with larynx preservation is the treatment of choice and, in cases with a local recurrence, a laryngectomy (TL) is performed as a salvage procedure. In more advanced stages or in cases of laryngeal dysfunction, a total laryngectomy (TL), with or without postoperative (chemo)radiotherapy is advised.^1^, ^2^

Since the thyroid gland is directly located in the field of laryngeal cancer treatment, thyroid gland dysfunction after radiotherapy or surgery for laryngeal or hypopharyngeal carcinoma of up to 88% have been reported, especially after (hemi)thyroidectomy during total TL.^3^, ^4^, ^5^, ^6^, ^7^, ^8^ Hypothyroidism is an irreversible condition, requiring lifelong thyroid hormone substitution. It is essential to recognize and treat the symptoms of hypothyroidism in an early stage especially because effective treatment is available.^9^, ^10^, ^11^ Previous studies on hypothyroidism as a consequence of radiotherapy or (hemi)thyroidectomy in the treatment of laryngeal or hypopharyngeal cancer, were performed before the introduction of intensity‐modulated radiotherapy or did not evaluate the impact of various combined treatment strategies used nowadays.^3^, ^4^, ^6^, ^12^

We hypothesized that if earlier radiotherapy has been affecting thyroid gland function, the additional partial removal of the thyroid gland during TL could result in a rapid onset of hypothyroidism. In patients treated with radiotherapy after TL without (hemi)thyroidectomy, one could expect a later or no onset of hypothyroidism. In the modern era of achieving larynx preservation, three patients groups at risk for the development of hypothyroidism after laryngectomy can be identified: (a) patients with a local recurrence after earlier organ preserving RT who have to be treated by salvage laryngectomy without additional radiotherapy (RT‐TL), (b) patients after TL followed by planned irradiation as primary treatment (TL‐RT), and (c) patients treated by salvage laryngectomy with a postoperative second period of irradiation (RT‐TL‐RT). Although adequate monitoring and early recognition of hypothyroidism has been advised, no data exists on the frequency and onset of developing hypothyroidism in these different patient groups.^3^, ^5^, ^6^, ^7^ In this study, we aimed to determine the likelihood and onset of developing hypothyroidism in patients after primary TL with postoperative RT, as well as in patients after salvage TL with or without second irradiation. Furthermore, we aimed to find independent predictive factors for the development of hypothyroidism. We also analyzed the consequences of a (hemi)thyroidectomy for the development of hypothyroidism in these three groups and calculated the euthyroid survival.

## 2 PATIENTS (OR SUBJECTS) AND METHODS

### 2.1 Patients

In this retrospective study, we included all 138 patients that underwent a TL at the University Medical Center Groningen, the Netherlands, between January 2010 and January 2018. Ten patients were excluded as in six cases the data on thyroid gland function was incomplete and four patients were excluded because they were using levothyroxine prior to their TL. In the remaining 128 patients, we retrospectively recorded the tumor site, tumor stage, radiotherapy regimen, concomitant chemotherapy, radiotherapy field size, the amount of thyroid surgery, thyroid gland function, and euthyroid survival.

### 2.2 Treatment regimens

Of all 128 patients, 17 patients received three‐dimensional conformal radiotherapy and 111 received intensity‐modulated radiotherapy (IMRT) and 23 patients additionally received chemotherapy. For initial larynx preserving radiotherapy, the irradiation schedule was 70 Gy in 35 × 2 Gy fractions, five times per week over 7 weeks and in cases of T1 N0 laryngeal carcinomas this was 66 Gy in 33 × 2 Gy fractions. Patients that received radiotherapy for a T1 or T2a laryngeal tumor were irradiated with a significantly smaller field.

An accelerated schedule of six fractions per week up to a total dose of 70 Gy was given in patients not eligible for chemoradiation, which was combined with cetuximab in three patients. Patients aged 70 and older years were treated solely with radiotherapy (ie, 70 Gy in 35 × 2 Gy fractions, five times per week for a period of 7 weeks). For the TL‐RT group the irradiation schedule was 66 Gy in 33 × 2 Gy fractions or 56 Gy in 28 × 2 Gy fractions depending on the tumor free margins. For the RT‐TL‐RT group, the second irradiation schedule was 60 Gy in 30 × 2 Gy fractions or 54 Gy in 36 × 1.5 Gy fractions twice a day. Chemotherapy consisted of 3 cycles carboplatin 300‐350 mg/m^2^ at day 1 in combination with 5‐Fluorouracil 600 mg/m^2^ as continuous infusion on days 1‐4 in 3‐week cycles. Cetuximab was given to patients younger than 70 years of age with locally advanced disease and a contraindication for chemotherapy. Cetuximab was started 1 week before start of radiotherapy with an initial dose of 400 mg/m^2^ followed by weekly doses of 250 mg/m^2^ during radiotherapy. A simultaneous integrated boost was used in patients receiving IMRT treatment. Most patients were treated with a bilateral elective irradiation of the neck to a total dose of 54.25 Gy in 35 × 1.55 Gy fractions. The primary tumor and the suspected lymph nodes were treated to a total dose of 70 Gy, in 35 × 2 Gy fractions. In the 52 patients who received thyroid gland surgery, a subtotal thyroidectomy was performed in four cases, a (hemi)thyroidectomy in 44 cases and a partial (hemi)thyroidectomy in four cases. In case of a hemithyroidectomy, effort was made not to damage the inferior and superior thyroid gland arteries of the contralateral thyroid gland.

### 2.3 Thyroid gland function

Levels of thyroid stimulating hormone (TSH), as well as free thyroxine (FT4) were assayed by electrochemiluminescence immunoassay (Roche Modular E170, Roche, Switzerland). TSH reference values established in healthy individuals were 0.5‐4.0 mU/L in our hospital. Thyroid gland function was evaluated by TSH and FT4 analyses periodically and retrospectively retrieved from the laboratory results section of the electronic patient files. Whenever applicable, the thyroid gland function values of the patients, until the moment they had developed hypothyroidism, were clustered in in the following time periods: prior to surgery, the week after surgery, 1‐2 weeks after surgery, 2‐4 weeks after surgery, 5‐8 weeks after surgery, 3‐6 months after surgery and subsequently every 6 months. In the case of multiple TSH samples, the mean value of the samples across this time period was used.

Patients were stated to have thyroid dysfunction when levothyroxine treatment was started because of clinical signs of hypothyroidism or when the TSH level exceeded 10.0 mU/L.

Time to onset of hypothyroidism was defined as the time (months) between TL and the moment hypothyroidism has developed in patients who were alive and developed hypothyroidism. Euthyroid survival additionally included information from patients without hypothyroidism and euthyroid survival time was calculated from TL until hypothyroidism (event), date of death (censored), or the last moment of follow‐up without hypothyroidism (censored). Patient follow‐up was last checked in January 2019.

### 2.4 Statistical analysis

Statistical analysis was performed using IBM SPSS Statistics 22 for Microsoft Windows (SPSS, Chicago, Illinois). Pearson's chi‐squared test and nonparametric Mann‐Whitney *U* test to analyze differences between groups. Euthyroid survival curves were constructed using the Kaplan‐Meier method and survival between groups were compared using the log‐rank test. After testing the proportional hazard assumption, a univariable Cox regression analysis on treatment regimen, tumor site, size of RT‐field, concomitant chemotherapy, performance of a neck dissection, hemithyroidectomy, gender, and age that could affect thyroid function was performed. Factors which were related to thyroid function and impacted the beta for treatment regimen, were included in the multivariable model. *P* values <.05 were considered statistically significant.

This study was approved by Institutional Ethical Review Board of the University Medical Center Groningen.

## 3 RESULTS

### 3.1 Risk of developing hypothyroidism after TL

Patient characteristics and clinical data are shown in Table 1. After a median follow‐up of 29.6 months (range:1.6‐99.5), hypothyroidism developed in 69 of the 128 (54%) patients (Table 2). In 60 of the 69 patients, hypothyroidism was diagnosed by TSH > 10.0 mU/L and in nine patients because of clinical signs of hypothyroidism in combination with increasing TSH levels.

**Table 1:** Demographic and clinical data of 128 studied patients with a TL in relation the development of hypothyroidism

		All patients (128 patients) n (%)	Hypothyroidism (69 patients) n (%)	No hypothyroidism (59 patients) n (%)
Gender	Male Female	113 (88.3) 15 (11.7)	58 (84.1) 11 (15.9)	55 (93.2) 4 (6.8)
Age	Median (years), (SD)	66, (9.3)	64, (8.7)	69, (9.4)
Location tumor	Hypopharynx	33 (25.8)	19 (27.5)	14 (23.7)
	Larynx	95 (74.2)	50 (72.5)	45 (76.3)
T Classification	T1	9 (7.0)	1 (1.4)	8 (13.6)
	T2a larynx T2b larynx	10 (9.4) 4 (3.1)	6 (8.7) 2 (2.9)	4 (6.8) 2 (3.4)
	T2 hypopharynx	12 (9.4)	4 (5.8)	8 (13.6)
	T3	33 (25.8)	18 (26.1)	15 (25.4)
	T4	60 (46.9)	38 (55.1)	22 (37.3)
Treatment regimen	RT‐TL TL‐RT RT‐TL‐RT	51 (39.8) 55 (43.0) 22 (17.2)	25 (36.2) 30 (43.5) 14 (20.3)	26 (44.1) 25 (42.3) 8 (13.6)
RT field	Larynx only Larger	16 (12.5) 112 (87.5)	6 (8.7) 63 (91.3)	10 (16.9) 49 (83.1)
(hemi)thyroidectomy	Not performed	76 (59.3)	31 (44.9)	45 (76.3)
	Performed	52 (40.6)	38 (55.1)	14 (23.7)
Neck dissection	Not performed	32 (25.0)	16 (23.2)	16 (27.1)
	Performed	96 (75.0)	53 (76.8)	43 (72.9)
Chemotherapy	No concomitant chemotherapy	105 (82.0)	57 (82.6)	48 (81.3)
	Concomitant chemotherapy with carboplatin/5‐FU	23 (18.0)	12 (17.4)	11 (18.7)

Abbreviations: 5‐FU, 5‐Fluorouracil; RT, radiotherapy; TL, total laryngectomy.

**Table 2:** Overview of number of TSH measurements in relation to number of patients who developed hypothyroidism

	Number TSH measurements in 59 euthyroid patients	Number of TSH measurements in 69 patients who developed hypothyroidism	total number of TSH measurements	Number of euthyroid patients (at risk)	Number of patients who developed hypothyroidism
Before TL	32	38	70	128	0
Perioperative	56	65	121	105	23
1‐3 months after TL	49	62	111	101	27
3‐6 months after TL	16	14	30	97	31
6‐12 months after TL	37	19	56	79	49
12‐18 months after TL	29	20	49	71	57
18‐24 months after TL	23	13	36	69	59
2‐3 years after TL	11	5	16	64	64
3‐4 years after TL	5	2	7	62	66
4‐5 years after TL	2	1	3	60	68
5‐6 years after TL	2	0	2	59	69
6‐7 years after TL	1	0	1	59	69

*Note*: Perioperative: 1 month before total laryngectomy until 1 month after total laryngectomy.

In eight of the nine patients thyroid gland substitution therapy was started because of increasing TSH levels; TSH levels in these eight patients ranged from 4.89 (after 2 weeks) till 9.95 (after 9 months). In the remaining patient, thyroid gland substitution therapy was started within 2 weeks after TL, because this patient had a nearly total thyroidectomy.

**Figure 1:**
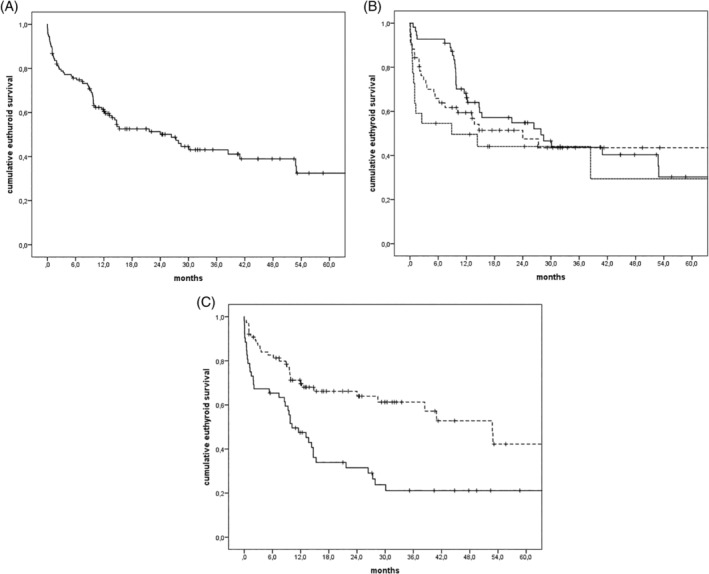
A, Cumulative euthyroid survival of all TL patients. B, Cumulative euthyroid survival stratified for treatment regimen: RT‐TL (‐‐‐), TL‐RT (—) and RT‐TL‐RT (···). C, Cumulative euthyroid survival in patients with (—) and without (‐‐‐) a (hemi)thyroidectomy

TSH was measured within 1 week after surgery in 68 cases. In 100 of 128 patients TSH was assessed either preoperatively or during the postoperative week and in 121 of the 128 patients TSH was measured within 4 weeks after TL. In the remaining seven patients, TSH was measured when signs of possible hypothyroidism developed. Hypothyroidism was confirmed in four out of these seven patients: in one patient after 7 months, in one patient after 9 months, in one patient after 10 months, and one patient 2 years after TL. Five years euthyroid survival was 32.5% and mean euthyroid survival was 26.3 months (in Figure 1A). Both univariable and multivariable analysis revealed (hemi)thyroidectomy as an independent risk factor for developing hypothyroidism after TL (Table 3). Patients who developed hypothyroidism were on average younger (mean: 63.1; SD: 8.7; range: 46‐84 years) than patients without hypothyroidism (mean: 68.0; SD: 9.4; range: 50‐91 years; *P* < .001). Multivariable analysis revealed a lower risk of developing hypothyroidism after planned TL followed by planned postoperative irradiation (hazard ratio [HR]: 0.49; 95% confidence interval [CI]: 0.28‐0.86) compared to salvage TL after radiotherapy (RT‐TL) (*P* = .013). There was an increased risk for developing hypothyroidism after salvage TL with a second period of irradiation (HR: 1.78; [95 CI: 0.91‐3.48], *P* = 0.091). Twelve out of 16 patients with an initial T1‐2a glottic cancer who received larynx only fields, were treated with salvage TL without postoperative radiotherapy. The risk of developing hypothyroidism in this group has not significantly decreased (HR: 0.69; [95 CI: 0.28‐1.17], *P* = .42), since five of these 12 patients developed hypothyroidism. Gender, tumor site, size of radiotherapy field, concomitant chemotherapy, and additional neck dissection were not associated with the risk of developing hypothyroidism.

**Table 3:** Univariable and multivariable Cox regression analysis for development of hypothyroidism

Variables		Univariable model		Multivariable model	
		HR (95% CI)	*P* value	HR (95% CI)	*P* value
Treatment regimen	RT‐TL TL‐RT RT‐TL‐RT	1 (reference) 0.81 (0.48‐1.39) 1.39 (0.72‐2.68)	.454 .327	1 (reference) 0.49 (0.28‐0.86) 1.78 (0.91‐3.48)	**.014** .090
Tumor site	Hypopharynx Larynx	1 (reference) 0.89 (0.52‐1.50)	.651		
RT field	Larynx only (one period) Larger field	1 (reference) 1.45 (0.58‐3.62)	.42		
Chemotherapy	No concomitant chemotherapy concomitant chemotherapy	1 (reference) 1.09 (0.59‐2.04)	.775		
Neck dissection	No additional ND Additional ND	1 (reference) 0.95 (0.54‐1.67)	.867		
(Hemi)thyroidectomy	Not performed Performed	1 (reference) 2.37 (1.47‐3.82)	**<.001**	1 (reference) 2.99 (1.81‐4.95)	**<.001**
Gender	Male Female	1 (reference) 1.51 (0.79‐2.89)	.208		
Age (median)	Continuous variable	0.95 (0.92‐0.98)	**<.001**	0.93 (0.91‐0.96)	**<.001**

Abbreviations: 95% CI, 95% confidence interval; HR, Hazard ratio; ND, neck dissection; RT, radiotherapy. Significant HRs and significant *P* values are marked in bold font.

### 3.2 Time of onset and risk of hypothyroidism after TL in relation to treatment regimen

**Figure 2:**
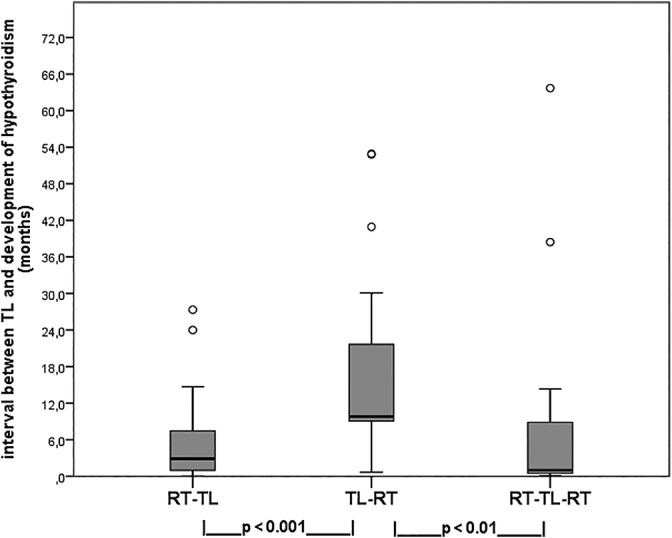
Boxplots of interval (in months) between TL and development of hypothyroidism in 69 patients in relation to the three treatment regimens (statistical analysis by Mann‐Whitney *U* test)

Although the interval between TL and the diagnosis of hypothyroidism showed substantial overlap among treatment groups due to the large variability, it was significantly longer (median: 9.8 months; mean: 15.9 months; 0.7‐52.9 months) in the TL‐RT treated patients compared to both the RT‐TL (median: 2.9 months; mean: 5.9 months; 0‐27.3 months; *P* < .001) and the RT‐TL‐RT (median: 1.0 months; mean: 9.5 months; 0.1‐63.7 months; *P* < .01) group (Figure 2). Intervals between the RT‐TL and RT‐TL‐RT group did not differ.

Of the 51 patients treated with *RT‐TL*, 25 patients (49.0%) developed hypothyroidism after median euthyroid survival of 24.0 months (95% CI: 7.6‐40.3 months) as shown in Figure 1B.

Thirty out of the 55 patients (54.5%) treated with *TL‐RT* developed hypothyroidism after a median euthyroid survival of 27.9 months (95% CI: 12.0‐43.7 months), as shown in Figure 1B.

Of the 73 patients treated with RT as a primary treatment, 22 were re‐irradiated after salvage TL *(RT‐TL‐RT)*. In 14 of these 22 patients (63.6%) hypothyroidism has developed after a median euthyroid survival of 8.9 months (95% CI: 0‐27.2 months).

### 3.3 The influence of thyroid gland surgery on the development of hypothyroidism in relation to treatment regimen

A hemithyroidectomy or subtotal thyroidectomy was performed during laryngectomy in 52 patients. Out of these 52 patients, 38 (73.1%) patients developed hypothyroidism during follow up. The median time to develop hypothyroidism for the group that underwent a (hemi)thyroidectomy was 8.0 months (mean: 8.5; SD 8.9; range: 0‐30.1 months), while in the population without a hemythyroidectomy the median time to develop hypothyroidism was 37.8 months (mean 35.5; SD 21.7; range 5.5‐80.0 months; *P* < .05). Median euthyroid survival was 10.2 months (95% CI: 5.6‐14.7 months) with (hemi)thyroidectomy and 52.8 months (95% CI: 36.5‐69.1 months) in the patients without (hemi)thyroidectomy (*P* < .001). Two years euthyroid survival was 24% in patients after (hemi)thyroidectomy and 61% in patients without a (hemi)thyroidectomy (Figure 1C) (*P* < .001). The risk of developing hypothyroidism after (hemi)thyroidectomy is lower in the TL‐RT group (*P* = .006) compared to a salvage TL with or without re‐irradiation (Table 4). In the patient group without thyroid gland surgery, the risk of hypothyroidism was not influenced by treatment regimen.

**Table 4:** Multivariable cox regression analysis on the risk of developing hypothyroidism in relation to treatment regimen and hemithyroidectomy

Variables		Hemithyroidectomy performed		Hemithyroidectomy not performed	
		HR (95% CI)	*P* value	HR (95% CI)	*P* value
Treatment regimen	RT‐TL TL‐RT RT‐TL‐RT	1 (reference) 0.36 (0.18‐0.75) 1.63 (0.64‐4.14)	**.006** .305	1 (reference) 0.80 (0.34‐1.86) 2.03 (0.76‐5.43)	.604 .161
Age (median)	Continuous variable	0.94 (0.90‐0.98)	**.004**	0.93 (0.88‐0.97)	**.001**

**Figure 3:**
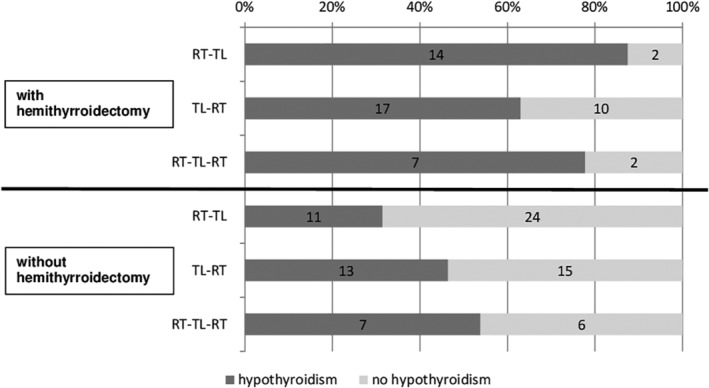
Schematic overview of the number of patients who developed hypothyroidism in relation to both (hemi)thyroidectomy and treatment regimen

In Figure 3, the relation between (hemi)thyroidectomy, treatment regimen and the development of hypothyroidism is illustrated. Hypothyroidism developed in 21/25 (84%) of the salvage TL patients (both RT‐TL and RT‐TL‐RT groups) in which a (hemi)thyroidectomy had to be performed. In 27 of the TL‐RT treated patients (49%), a (hemi)thyroidectomy was performed and 17 of these 27 patients (63%) developed hypothyroidism. In the patients without thyroid gland surgery, 13/28 (46%) of the TL‐RT patients and 18/48 (38%) of the salvage TL patients developed hypothyroidism.

## 4 DISCUSSION

Thyroid dysfunction after treatment for laryngeal or hypopharyngeal carcinoma is a well‐known complication after TL in combination with radiotherapy.^7^ In our study, 54% of the patients developed thyroid gland dysfunction, which is in line with earlier studies.^3^, ^4^, ^5^, ^6^, ^7^, ^8^, ^13^ However, in daily clinical care the appropriate time and interval to evaluate thyroid gland function after TL remains unclear. Unrecognized subclinical hypothyroidism might therefore gradually progress. Especially shortly after intensive oncological treatment, other side effects of the oncological treatment could mimic the first signs of thyroid gland dysfunction. This is the first study analyzing the risk for hypothyroidism in relation to the various treatment regimens in which a TL has been performed.

We identified TL‐RT as a treatment regimen associated lower risk (HR 0.49) for developing hypothyroidism and with a prolonged time window in which hypothyroidism develops. Hypothyroidism developed 6 to 10 months later in the patients treated with a primary TL followed by RT. A planned TL followed by irradiation has a lower risk (HR: 0.49) of developing hypothyroidism as compared to TL as a salvage procedure, especially if this salvage TL has to be followed by another period of radiotherapy (HR: 1.78). In patients in with a salvage TL, the smaller interval between TL and onset of hypothyroidism could be explained by the major influence of irradiation on thyroid gland function on the long term, since irradiation was administered months, sometimes years, before definitive salvage TL. The influence of radiotherapy on the onset of hypothyroidism in head and neck patients with cancer in general has been studied extensively, but our study focused on the three main treatment strategies involving laryngectomized patients.^4^ Euthyroid survival in TL‐RT treated patients was better during the first months after TL, but no statistical significant differences in euthyroid survival was detected between the different treatment strategies. This could be explained by the large variance in both groups and the probability that also cancer survival itself influenced euthyroid survival.

We found that (hemi)thyroidectomy during TL is the most important independent risk factor responsible for almost tripling (HR: 2.98) the of developing hypothyroidism after TL compared to patients without a (hemi)thyroidectomy. Furthermore, in cases in which a (hemi)thyroidectomy had to be performed, we did find a significantly higher incidence in thyroid gland dysfunction, as shown in other studies.^6^, ^14^, ^15^ Lo Galbo et al analyzed 137 patients with laryngeal or hypoharyngeal cancer treated with surgery and/or radiotherapy.^6^ Using multivariate analysis, they also found that hemithyroidectomy is strongly associated with the development of hypothyroidism, but did not weigh the influence of treatment regimen. Although a median time of 10 months for the development of hypothyroidism was described in this study, no information was provided on euthyroid survival after TL since only 37 patients (27% of the studied population) were treated with TL. The major influence of previous irradiation in (hemi)thyroidectomized patients is reflected by different risks for in the development of hypothyroidism between the patients who already were irradiated (RT‐TL and RT‐TL‐RT group), compared to TL‐RT patients with a HR of only 0.36 (95% CI: 0.18‐0.75) (Table 4). Hemithyroidectomy as a routine procedure when performing a TL is obsolete. In our institute, a hemithyroidectomy is only performed in advanced laryngeal or hypopharyngeal carcinoma with an increased risk of involvement of the thyroid gland as advised by Mendelsohn.^16^

The finding after multivariable analysis that hypothyroidism after TL was weakly associated with younger age was surprising, but might be explained by a selection bias: TL could have been performed more frequently in the younger age group, as has been described in an earlier study, whereas the slow progression of impaired thyroid gland function in older patients is affected by a worse overall survival in these advanced tumors.^17^ Future studies should clarify these findings.

Because the measurement of thyroid gland function in our institute is part of standard follow‐up, we were able to analyze these retrospectively from routinely collected data. There are several limitations in this study. First limitation is that not all the patients adhere to the standard protocol and sometimes thyroid gland function was determined additionally or omitted. A second limitation is that the effect of the radiotherapy doses on the thyroid gland could not be calculated, since it is difficult to adjust for removal of the thyroid gland during salvage surgery or double irradiation in the RT‐TL‐RT group. Due to the relatively low number of patients with 5 years euthyroid survival, we cannot provide evidence for the duration of monitoring thyroid gland function after TL. Although the retrospective character of this study is another limitation (eg, operation reports did not give reliable information regarding exact preservation and avoiding electrocoagulation near the thyroid gland vessels) no previous studies tried to differentiate between treatment regimens considering the risk and moment of developing hypothyroidism in laryngectomized patients.

We identified TL‐RT as the regimen with a longer interval and the lowest chance for development of hypothyroidism, even after (hemi)thyroidectomy. We revealed that hypothyroidism will develop later (median difference of 7 months) in patients treated with TL and postoperative RT than in patients treated with a salvage TL after earlier RT. We can also conclude from this study that the onset of hypothyroidism varies widely, eventually up to 70% of the patients who are still alive 5 years after TL develop hypothyroidism and the mean euthyroid survival is comparable across all treatment groups.

Although we cannot provide a solid guideline for the most appropriate moment for measuring thyroid gland function, this study could help in daily clinical practice by making head and neck oncologists aware that a patient with double irradiation could develop hypothyroidism as late as 3 years after TL and patients treated with primary TL could develop hypothyroidism before postoperative radiotherapy has even started.

Therefore, we advise to evaluate thyroid gland function not only during the first weeks after TL, but at least every 6 months for at least 4 years after TL and to be aware of a later onset of hypothyroidism in the TL‐RT treated patients.

## 5 CONCLUSION

The risk of hypothyroidism is lower and the interval between TL and thyroid gland dysfunction is longer in patients with initial TL followed by postoperative radiotherapy, even after (hemi)thyroidectomy, as compared to salvage TL with or without a second irradiation. Thyroid gland surgery was additionally confirmed as an important predictive variable for the development of hypothyroidism.
